# Apoptotic Inducement of Neuronal Cells by Aluminium Chloride and the Neuroprotective Effect of Eugenol in Wistar Rats

**DOI:** 10.1155/2020/8425643

**Published:** 2020-01-27

**Authors:** Samuel Bolaji Mesole, Okpanachi Omachonu Alfred, Uthman Ademola Yusuf, Lwiindi Lukubi, Dailesi Ndhlovu

**Affiliations:** ^1^Department of Human Anatomy, Texila American University, Zambia; ^2^Department of Human Physiology, Kampala International University, Uganda; ^3^Department of Human Anatomy, Mulungushi University, Zambia; ^4^Department of Physiological Sciences, University of Zambia, Zambia; ^5^Department of Human Anatomy, Levy Mwanawasa Medical University, Zambia

## Abstract

Aluminium is known to accelerate oxidative stress, amyloid beta (A*β*) deposition, and plaque formation in the brain of rats. *Objective*. The present study is aimed at studying the neuroprotective effects of eugenol following aluminium-induced neurotoxicity on caspase-3, apoptotic proteins (Bcl-2 and Bax), and oxidative stress markers in Wistar rats such as superoxide dismutase (SOD), glutathione peroxidase (GPx), nitric oxide (NO), and assay oxidative stress to mitochondrial DNA (mtDNA) by measuring the levels of 8-hydroxy-2-deoxyguanosine (8-OHdG). *Materials and methods*. Twenty (20) adult Wistar rats were randomly divided into four (4) groups with five animals in each group. Route of administration was oral throughout the duration of this study and this study lasted for 21 days. Rats were sacrificed 24 hours after administration of the last dose (i.e., day 22) with 0.8 mg/kg ketamine as an anaesthetic agent. *Results*. Exposure to AlCl_3_ resulted in a significant (*p* < 0.01) elevation in the levels of nitric oxide and 8-hydroxy-2-deoxyguanosine (8-OHdG), enhanced the activity of caspase-3, increased the level of proapoptotic protein Bax and reduced the levels of antiapoptotic protein Bcl-2, and significantly (*p* < 0.01) reduced the levels of SOD and GPx. However, treatment with eugenol resulted in a significant reduction (*p* < 0.01) in the levels of nitric oxide (NO) and 8-hydroxy-2-deoxyguanosine (8-OHdG) levels, inhibited the activity of caspase-3, increased levels of Bcl-2 and significantly (*p* < 0.05) reduced levels of Bax protein, respectively, and also significantly (*p* < 0.05) increased the levels of SOD and GPx. Our results would hereby suggest that eugenol would provide a therapeutic value against aluminium-induced oxidative stress as related to antioxidant and antiapoptotic activities.

## 1. Background Study

Aluminium (Al) is ever present in our environment. As a result, food is the major source for aluminium intake under physiological conditions [1]. The widespread presence of aluminium, both in the environment and in food, makes it almost impossible to avoid exposure to this metal ion [2]. A link between aluminium neurotoxicity and excitotoxicity was described by [3]. While aluminium, under certain conditions, can act as a prooxidant. However, aluminium appears to react with the superoxide radical, thus facilitating its destructive potential [3]. Experimental observations also indicate that formation of the superoxide plays a major step in contributing to excitotoxicity-mediated neuronal death through generation or production of peroxynitrite [4]. One of the primary destructive reactions in the excitotoxic cascade is the building up of peroxynitrite which is formed when increased or elevated levels of superoxide react with nitric oxide (NO), which is also produced during excitotoxicity [3]. Aluminium could also enhance excitotoxicity by inducing apoptosis of astrocyte. Astrocytes are thought to be a primary target of aluminium accumulation among neuronal cells [5]. Aluminium has been shown to drastically lower neuronal reduced glutathione levels, and astrocytes are the major source of glutathione for neurons [6].

Increased levels of glutamate may also inhibit glutathione production intracellularly by inhibiting the cystine/glutamate antiporter [7].

Eugenol (IUPAC name is 4-allyl-2-methoxyphenol), having a molecular formula of C_10_H_12_O_2_ with a molecular weight of 164.21, is mainly found in clove oil, camphorated oil, cinnamon leaf oil, and nutmeg oil. The anticancer property of eugenol has become paramount in recent years. Synthetically produced anticancer drugs have some toxic side effects and may eventually cause potential damage to normal neuronal cells within the nervous system. Eugenol therefore has a better application potential in the prevention and treatment of some type of cancers. Eugenol prevents apoptosis of Bcl-2, COX-2, and IL-1*β*, hence reducing inflammation and death of neurons [8].

The aim of this study is to investigate the neuroprotective effect of eugenol on aluminium-induced neurotoxicity. This type of study will explore the potential therapeutic and preventive approaches which can be used to minimize the destructive effects of aluminium to the brain.

## 2. Materials and Methods

### 2.1. Chemicals and Experimental Animals

Aluminium chloride (AlCl_3_) used for this study was purchased from Guangdong Guanghua Sci-Tech Co. Ltd., Shantou, Guangdong, China, and manufactured by Yueyang Jiazhiyuan Biological Co. Ltd., China. Eugenol used for this study was purchased from Wuhan JCJ Logis, China, and manufactured by Yueyang Jiazhiyuan Biological Co. Ltd., China.

A total of 20 adult Wistar rats was used for this study with an average weight of 160 g were obtained from the Animal House of the Department of Human Anatomy, School of Medicine, Texila American University, Lusaka, Zambia, and they were acclimatized for two weeks prior to the commencement of the experiments. All rats were given food (rat chow-tiger feeds) and water *ad libitum* and in strict adherence to Texila American University Research and Ethics committee (TAUREC). Treatment groups were administered eugenol/aluminium chloride in addition to water and rat chow.

### 2.2. Experimental Protocol

To study the neuroprotective effects of eugenol following aluminium-induced neurotoxicity, rats were randomly divided into four (4) groups with five (5) animals in each group. Group I was administered 150 mg/kg (5% LD_50_) of eugenol; Group II, 150 mg/kg eugenol and 100 mg/kg aluminium chloride (AlCl_3_); Group III, 100 mg/kg aluminium chloride (AlCl_3_) [9]; and Group IV, 2 mg/kg of distilled water as placebo for a duration of twenty-one (21) days. All administration was via oral route throughout the duration of this study.

Twenty-four (24) hours after last administration (i.e., day 22), the animals were humanely sacrificed using 0.8 mg/kg ketamine as an anaesthetic agent. The rat brains were carefully removed and stripped of any adhering structures and washed twice in ice-cold 50 mM Tris-HCl with pH 7.4. The rat brain was weighed using a sensitive digital weighing balance (#FA2004 manufactured by WANT Balance Co., Ltd.) and homogenized to yield a 10% (*w*/*v*) homogenate in an ice-cold medium. These contained 50 mM Tris-HCl, pH 7.4. The homogenates obtained was centrifuged at the rate of 1200 g for 10 minutes (600 seconds) at 4 degree Celsius. The supernatants obtained were used for various protein and enzymatic estimations. The total protein content of the homogenate was assayed by the method of [10] which was reported by [11] using bovine serum albumin as a yardstick.

### 2.3. Oxidative Stress Markers

Superoxide dismutase (SOD) activity was investigated with rat SOD ELISA Kit (supplied by WKEA Med Supplies Corp., China, and manufactured by Fine Test China) and used as stated by the manufacturer. The procedure was based on the method of [12] as reported by [13]. Glutathione peroxidase (GPx) activity was measured using GPx ELISA Kit (supplied by WKEA Med Supplies Corp., China, and manufactured by Fine Test china) and used as stated by the instructions of the manufacturer. The procedure was based on the method of [14]. These levels were expressed as *μ*mol/ml.

Intramitochondrial accumulation of 8-OHdG was used as a measure of mtDNA oxidative stress using the 8-OHdG ELISA Kit (supplied by WKEA Med Supplies Corp., China, and manufactured by Fine Test china). Mitochondrial DNA (mtDNA) was isolated using the method of [15] as reported by [16].

### 2.4. Determination of Apoptotic Markers

Brain homogenates were made in lysis buffer and these brain homogenates were analyzed using a colorimetric caspase-3 assay kit (supplied by WKEA Med Supplies Corp., China, and manufactured by Fine Test China); these assay kits was used with strict compliance to the manufacturer's instructions. Bcl-2 protein and Bax protein levels were measured/assayed in brain tissue lysates by the appropriate ELISA Kit (supplied by WKEA Med Supplies Corp., China, and manufactured by Fine Test china), and the procedure for the assay was performed according to the manufacturer's instructions, and results were expressed as ng/mg.

### 2.5. Statistical Analysis

Results obtained from this research were analyzed with the statistical package for social sciences (IBM SPSS version 21.0, SPSS) and Microsoft Office Excel 2007 for charts. These results were expressed as mean ± standard error of mean (SEM) and significant differences among means of the groups were determined with one-way analysis of variance (ANOVA) with Tukey's *post hoc test* for significance. Paired sample *t*-test was used for comparison of mean. Values were considered significant when *p* ≤ 0.05.

## 3. Results

**Figure 1:**
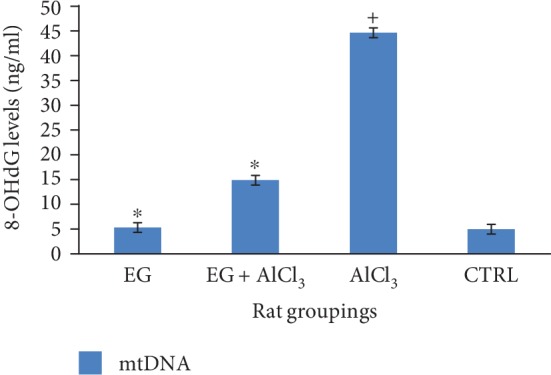
Effect of eugenol on brain mtDNA levels following administration of aluminium chloride on Wistar rats. *n* = 4; mean ± SEM; one way Tukey's post hoc test: ^∗^*p* < 0.01 when compared with the aluminium-treated group and ^+^*p* < 0.001 when the AlCl_3_ group is compared to the control.

Figure 1 below shows the effect of eugenol on levels of 8-OHdG following the administration of aluminium chloride. This result shows a significant (*p* < 0.01) increase in the levels of 8-OHdG in the group administered with AlCl_3_ when comparison is made to other groups. Treatment with eugenol however resulted in a significant (*p* < 0.01) reduction in the levels of 8-OHdG when compared to the aluminium-treated group.

**Figure 2:**
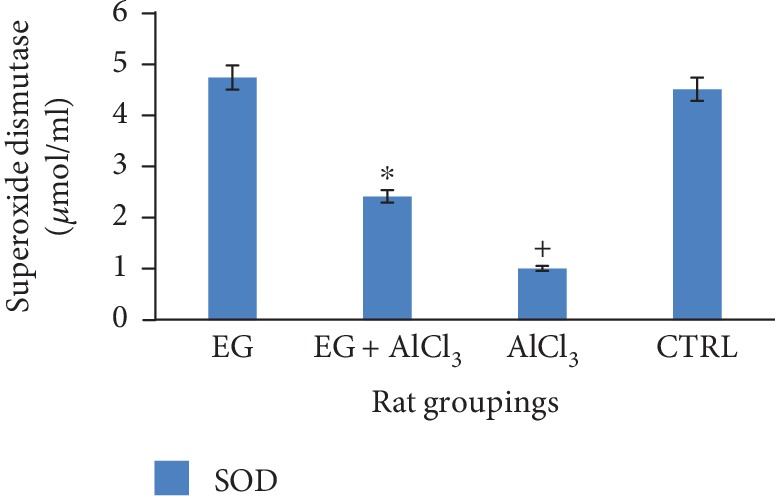
Effect of eugenol on brain SOD levels following administration of aluminium chloride on Wistar rats. *n* = 4; mean ± SEM; one-way Tukey's post hoc test: ^∗^*p* < 0.05 when compared with the aluminium-treated group and ^+^*p* < 0.01 when the Alcl_3_ group is compared to the control.

Figure 2 below shows the effect of eugenol on brain superoxide dismutase (SOD) levels following administration of aluminium chloride. This result revealed a significant (*p* < 0.01) reduction in the tissue activity of SOD when comparison is made to the control. However, treatment with eugenol revealed a significant (*p* < 0.05) increase in the tissue activity of SOD (expressed as *μ*mol/ml).

**Figure 3:**
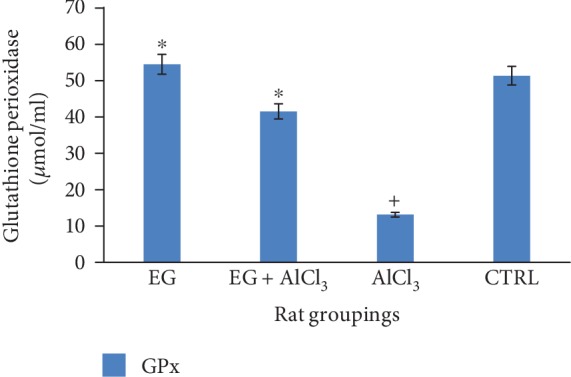
Effect of eugenol on brain GPx levels following administration of aluminium chloride on Wistar rats. *n* = 4; mean ± SEM; one-way Tukey's post hoc test: ^∗^*p* < 0.05 when compared with the aluminium-treated group and ^+^*p* < 0.01 when the AlCl_3_ group is compared to the control.

Figure 3 below shows the effect of eugenol on GPx levels in the brain following administration of aluminium chloride. This result revealed a significant (*p* < 0.01) reduction in the activity of GPx when the aluminium-treated group is compared to the control. However, eugenol treatment significantly (*p* < 0.05) increased brain GPx levels when compared to the aluminium-treated group (expressed as *μ*mol/ml).

**Figure 4:**
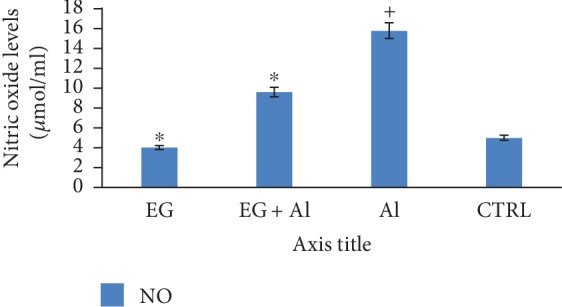
Effect of eugenol on brain nitric oxide (NO) levels following administration of aluminium chloride on Wistar rats. *n* = 4; mean ± SEM; one-way Tukey's post hoc test: ^∗^*p* < 0.05 when compared with the aluminium-treated group and ^+^*p* < 0.05 when the AlCl_3_ group is compared to the control.

Figure 4 below shows the effect of eugenol on brain levels of nitric oxide (NO) (expressed as *μ*mol/ml) following administration of aluminium chloride. This result showed a significant (*p* < 0.05) increase in the levels of nitric oxide within the brain tissue when the aluminium-treated group is compared to the control. However, treatment with eugenol revealed a significant (*p* < 0.05) reduction in brain levels of nitric oxide (NO) when the eugenol-treated group is compared with the aluminium-treated group.

**Figure 5:**
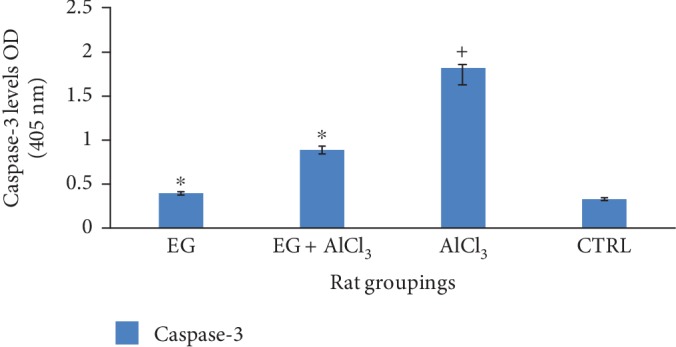
Effect of eugenol on brain caspase-3 levels following administration of aluminium chloride on Wistar rats. *n* = 4; mean ± SEM; one way Tukey's post hoc test: ^∗^*p* < 0.05 when compared with the aluminium-treated group and ^+^*p* < 0.001 when the AlCl_3_ group is compared to the control.

Figure 5 below shows the effect of eugenol on the brain level of caspase-3 following administration of aluminium chloride. This result revealed a significant (*p* < 0.05) increase in the level of caspase-3 when compared to the control. However, treatment with eugenol reduced (*p* < 0.05) the level of caspase-3 within the brain.

Table 1 below shows the effect of eugenol on proapoptotic and antiapoptotic proteins following aluminium-induced neurotoxiciy in rats. This result revealed a significant (*p* < 0.05) increase in proapoptotic (Bax) levels, and this was significantly (*p* < 0.05) reduced upon treatment with eugenol. Antiapoptotic protein (Bcl-2) levels were significantly reduced in the aluminium-treated group while treatment with eugenol significantly (*p* < 0.05) increased the brain levels of Bcl-2.

## 4. Discussion

Several studies revealed that reductions in neuronal energy can dramatically amplify excitotoxic injury. Disruption of the mitochondrial function and energy metabolism by aluminium can be expected to result in a high degree increase in the sensitivity of neuronal cells to excitotoxicity and accelerate neuronal damage within the regions of the brain such as the cerebrum and cerebellum [17–19]. Metals such as aluminium and mercury are known as major environmental pollutants. These toxic metals are regarded as nonbiodegradable due to the fact that they cannot be broken down (degraded) by cells. They therefore constitute major global health risk due to their ability to accelerate or contribute to a variety of diseases [20].

Antioxidants have proven to be useful in conditions associated with oxidative stress [21, 22]. Oxidative stress which occurs due to the imbalance between production and detoxification of reactive oxygen species (ROS) has also been implicated in many neurodegenerative diseases [23, 24].

Aluminium-intoxicated rats show various markers or indicators of oxidative stress, and these can be expressed as elevated nitric oxide levels which in turn leads to the production and generation of ROS [25–27].

Superoxide dismutase is a potent endogenous enzymatic scavenger which can counterbalance the oxidative destruction brought about by the generation of free radicals [28, 29]. In this study, there was a reduction in the activities of superoxide dismutase. The reduction in its activity levels may be a result of its ability to clear free radicals within these cells. This indicates an increased free radical production as shown in Figure 2. The antioxidative capacity of these cells was hereby interrupted by aluminium chloride.

Aluminium can induce the production of nitric oxide in the microglia and astrocytes of the rat brain [30]. The increase in nitric oxide may be a result of the ability of aluminium chloride to accelerate the expression of iNOS. The present study revealed an increase in nitric oxide production as shown in Figure 4, thus indicating that aluminium-induced toxicity can result in changes in nitric oxide generation [31]. Eugenol inhibited lipopolysaccharide- (LPS-) dependent generation of nitric oxide, which can be attributed to the inhibition of protein synthesis of inducible nitric oxide synthase (iNOS) [32].

Oxidative damage is mediated by generation of free reactive oxygen species; therefore, the status of endogenous antioxidant enzymes under different treatment conditions was necessary [33]. Treatment with aluminium chloride resulted in a significant (*p* < 0.05) decrease in the activity of antioxidant enzymes like glutathione peroxidase and superoxide dismutase when compared to the control group as shown in Figures 2 and 3, respectively; this is in tandem with a previous study as reported by [26, 33]. However, administration of eugenol resulted in the increase in the activity of glutathione peroxidase and this is also in agreement with the reports of [34].

The generation of free radicals can result in damage to proteins and DNA [35]. Free radical (reactive oxygen species)-mediated effects on DNA can result to mutations of mitochondrial DNA, and hence damage to mitochondria DNA was assessed in this study.

Mitochondrial DNA (mtDNA) is highly susceptible to oxidative stress through the generation of reactive oxygen species; hence, the generation or production of reactive oxygen species can be very toxic to mitochondrial DNA [36, 37]. Various agents can trigger the release of various indicators of oxidative stress. These can all result in damage to DNA, and among these types of DNA damage caused by reactive oxygen species (ROS), generation of 8-hydroxy-2-deoxyguanosine (8-OHdG) is the most common one [38]. Therefore, the levels of 8-hydroxy-2-deoxyguanosine generated within the mtDNA can be used as a marker or an indicator of the level of cellular oxidative damage. Aluminium chloride in this study resulted in a significant increase in the levels of 8-OHdG as shown in Figure 1, which is in agreement with the study carried out in [39]. However, treatment with eugenol resulted in the reduction in the cellular level of 8-OHdG conferring a neuroprotective effect against reactive oxygen species- (ROS-) induced oxidative damage to mitochondrial DNA (mtDNA).

Exploring the mechanism by which eugenol attenuates aluminium chloride-induced apoptosis, proapoptotic (Bax) and antiapoptotic (Bcl-2) and caspase-3 protein levels were assayed in Wistar rat brain homogenates. Reactive oxygen species increase the permeability of the mitochondrial membrane and therefore results in mitochondrial failure [40]. The membrane of the mitochondria permeability is dependent upon the level of mitochondrial permeability transition pore; these results in the release of cytochrome c from within the inner mitochondrial membrane into the cytosol within the cytoplasm [41]. Once cytochrome c is released from within the mitochondrial membrane, cytochrome c then binds to Apaf-1 within the cytoplasm; these results in a complex that will activate caspase-9, and hence activation of apoptosis-inducing caspase-3 [42]. In this study, we observed aluminium chloride-induced apoptosis within the brain of Wistar rats via the activation of apoptotic caspase-3. Treatment with eugenol decreased the activity of caspase-3 as shown in Table 1.

These results suggest that eugenol might exert its antiapoptotic effects, by inhibiting or preventing caspase cascade activation.

Mitochondria-mediated intrinsic pathway is regulated by the proapoptotic (Bcl-2) group of proteins. The Bcl-2 protein family can be classified mainly into two subclassifications which is according to structural homology, and these include the antiapoptotic proteins and apoptosis-inducing proteins. The balance between the proapoptotic and antiapoptotic proteins is pivotal in determining the fate of a cell. Bcl-2 may function to negate damaging effects of ROS by reducing the levels of different ROS within the cytosol [43]. Bcl-2 was discovered to inhibit the release of cytochrome c within the mitochondrial membrane. In contrast to Bcl-2, Bax on the other hand monitors apoptosis, by controlling the release of cytochrome c into the cytosol resulting in caspase-3 activation [44]. The result from this study reveals that aluminium chloride resulted in high levels of proapoptotic protein Bax and lower levels of antiapoptotic protein Bcl-2. These results however also revealed that eugenol treatment significantly reversed the altered Bcl-2 and Bax levels induced by aluminium chloride which substantially restored the Bcl-2/Bax ratio. In this present research, eugenol administration inhibited all toxic events induced by aluminium chloride. Eugenol scavenges oxygen- and nitrogen-based reactants generated within the mitochondria, hence stabilizing the mitochondrial membrane and enhances and promotes antiapoptotic signaling.

Other natural products such as moringa leave extracts, clove extracts, and Psidium guajava leave extracts have been proven to be very potent antioxidants by inhibiting the generation of ROS and iNOS; these can all promote apoptosis within a cell [45]. Results obtained from the study with the abovementioned plant extracts were comparable to eugenol antioxidant effects as deduced from the present study.

Metals, such as lead (Pb), mercury (Hg), and zinc (Zn), are well-established models in the cognitive decline associated with neurodegenerative diseases as well as loss of neurites and formation of amyloid beta proteins. The deleterious effect of these metals with regard to generation of ROS can be compared to that of aluminium within biological systems [46].

## 5. Conclusion

In summary, exposure to aluminium chloride can result in the generation of free radicals, which resulted in the elevation of nitric oxide levels and reduction in the enzymatic and nonenzymatic antioxidant components. However, eugenol could protect against aluminium-induced neurotoxicity. This protective properties of eugenol may be due to the radical scavenging activity which maintains an appropriate level of nonenzymatic and enzymatic antioxidant fortification in opposition to aluminium chloride-induced neurotoxicity. These novel results could suggest that eugenol may be an effective treatment in attenuating the devastating and deteriorating effects of aluminium-induced neurotoxicity.

## Figures and Tables

**Table 1:** Effects of eugenol on proapoptotic and antiapoptotic proteins following aluminium-induced neurotoxicity in rats.

Parameter	EG	EG+AlCl_3_	AlCl_3_	CTRL
Bcl-2 (ng/mg)	15.25 ± 0.21^∗^	9.02 ± 0.45^∗^	5.12 ± 0.12^**+**^	17.06 ± 062
Bax (ng/mg)	2.41 ± 0.82^∗^	12.21 ± 0.71^∗^	19.52 ± 0.60^**+**^	3.21 ± 0.92
Bcl-2/Bax	6.32 ± 0.01^∗^	0.74 ± 0.01^∗^	0.26 ± 0.03^**+**^	5.31 ± 0.02

*n* = 4; mean ± SEM; one-way Tukey's post hoc test: ^∗^*p* < 0.05 when compared with the aluminium-treated group and ^+^*p* < 0.05 when the AlCl_3_ group is compared to the control.

## Data Availability

The data used to support the findings of this study are included within the article.
